# A dataset of gridded precipitation intensity-duration-frequency curves in Qinghai-Tibet Plateau

**DOI:** 10.1038/s41597-024-04362-1

**Published:** 2025-01-02

**Authors:** Zhihui Ren, Yan-Fang Sang, Peng Cui, Fei Chen, Deliang Chen

**Affiliations:** 1 Key Laboratory of Water Cycle & Related Land Surface Processes, Institute of Geographic Sciences and Natural Resources Research, Chinese Academy of Sciences, Beijing, 100101 China; 2 University of Chinese Academy of Sciences, Beijing, 100049 China; 3 Yarlung Zangbo Grand Canyon Water Cycle Monitoring and Research Station, Tibet Autonomous Region, Linzhi, 860000 China; 4 Key Laboratory of Compound and Chained Natural Hazards, Ministry of Emergency Management of China, Beijing, 100085 China; 5 Key Laboratory of Land Surface Pattern and Simulation, Institute of Geographic Sciences and Natural Resources Research, Chinese Academy of Sciences, Beijing, 100101 China; 6 POWERCHINA Chengdu Engineering Corporation Limited, Chengdu, 610072 China; 7 Department of Earth System Science, Tsinghua University, Beijing, 100084 China; 8 Department of Earth Sciences, University of Gothenburg, Gothenburg, 40530 Sweden

**Keywords:** Hydrology, Natural hazards

## Abstract

The Qinghai-Tibet Plateau (QTP), a high mountain area prone to destructive rainstorm hazards and inducing natural disasters, underscores the importance of developing precipitation intensity-duration-frequency (IDF) curves for estimating extreme precipitation characteristics. Here we introduce the Qinghai-Tibet Plateau Precipitation Intensity-Duration-Frequency Curves (QTPPIDFC) dataset, the first gridded dataset tailored for estimating extreme precipitation characteristics in QTP. The generalized extreme value distribution is chosen to fit the annual maximum precipitation samples at 203 weather stations, based on which the at-site IDF curves are estimated; then, principal component analysis is done to identify the southeast-northwest spatial pattern of at-site IDF curves, and its first principal component gives a 96% explained variance; finally, spatial interpolation is done to estimate gridded IDF curves by using the random forest model with geographical and climatic variables as predictors. The dataset provides precipitation information within 1, 2, 3, 6, 12, 24 hours and 5, 10, 20, 50,100 return years, with a 1/30° spatial resolution. The QTPPIDFC dataset can solidly serve for hydrometeorological-related risk management and hydraulic/hydrologic engineering design in QTP.

## Background & Summary

High mountain areas frequently encounter dangerous threats from natural disasters due to their steep terrain and extreme climatic conditions^1^. The Qinghai-Tibet Plateau (QTP), well-known as “The Third Pole” on earth, is a typical high mountain area that sustains billions of people locally and in its downstream areas^2^. QTP is also a high-hazard region suffering from frequent rainstorm hazards and their induced natural disasters, including flash floods, mudslides, and landslides, making it a global hotspot for the research of mountain natural disasters^3,4^. Extreme precipitation is one major driving factor of natural disasters in QTP^5^, where the interplay between rugged terrain and moisture transport facilitates the generation of extreme precipitation events^6^. Under the control of South Asia monsoon system, there is a high incidence of rainstorm events during summer period in the region^7,8^, triggering flash flood disasters within a short duration but strong intensity. These floods result in severe casualties and economic losses, accompanied by extensive damage to buildings, farms, roads, and other property^9,10^. Confronted with increasing flood susceptibility in QTP due to climate change^10^, investigating extreme precipitation characteristics is therefore of marked importance for mitigating and controlling natural disasters, as well as supporting hydraulic/hydrologic design and risk management strategies in the region.

Precipitation intensity-duration-frequency (IDF) curves afford a feasible approach to quantitatively describe extreme precipitation characteristics and have been widely applied in hydrometeorological risk management^11–14^. They graphically represent the relationship among intensity, duration, and the occurring probability of precipitation, providing a solid foundation for the research of rainstorm-related hazards, as well as the design of hydraulic infrastructures and drainage systems such as sewers, drains, dikes, dams, and bridges. The derivation of precipitation IDF curves is closely based on precipitation observations at representative stations. However, extreme precipitation characteristics indicate high spatial inhomogeneity in QTP, while the sparse distribution of limited rainfall stations causes difficulty in choosing representative stations and precipitation observations, further complicating the derivation of precipitation IDF curves for the whole QTP. As a result, accurately deriving precipitation IDF curves in QTP is a significant research gap.

Spatial interpolation is an alternative approach for deriving precipitation IDF curves at regional scales. A simple method is using conventional techniques (e.g., Kriging interpolation, inverse distance weighted method) to spatially interpolate the parameters in IDF curves^15–18^, however, it usually underestimates the spatial variability of IDF curves controlled by geographical and climatic conditions. Another feasible method is to establish the relationship between precipitation IDF curves and their influencing factors. For example, some studies tried to establish the relationship between geographical conditions^19,20^, mean annual precipitation^21,22^, rainstorm characteristics^23^ and surface topography^24^ and the spatial distribution of IDF curves. However, determining these relationships is difficult, as precipitation IDF curves always contain rich information with various time durations (e.g., from minutes to hours) and return periods. In recent years, some studies reported that the principal component analysis (PCA) method can effectively identify the dominant spatial pattern of precipitation IDF curves at regional scales^25,26^, and the identified spatial pattern can be further explained using suitable regression models with geographical and climatic variables as predictors^27^. Thus, the PCA method provides a more robust way for deriving precipitation IDF curves from stations to regional scales.

In this study, we focus on QTP as the study area and aim to generate gridded precipitation IDF curves for the entire region. Considering the limited data availability of extreme precipitation, we derive the IDF curves from stations to the entire QTP using the PCA method. Specifically, we choose a suitable probability distribution to fit the annual maximum precipitation data samples, based on which the at-site IDF curves are generated. After that, we apply the PCA method to identify the spatial pattern and the leading principal components (PCs) of at-site IDF curves, and further make a spatial interpolation following the regression-based relationship between PCs and geographical and climatic factors. As a result, the gridded dataset, called the Qinghai-Tibet Plateau Precipitation Intensity-Duration-Frequency Curves (QTPPIDFC), is generated. The QTPPIDFC dataset provides both the mean and coefficient of variance (*C*_*v*_) of gridded precipitation IDF curves with a 1/30° spatial resolution, serving hydrometeorological risk management and hydraulic engineering design in QTP.

## Methods

### Overview

**Fig. 1:**
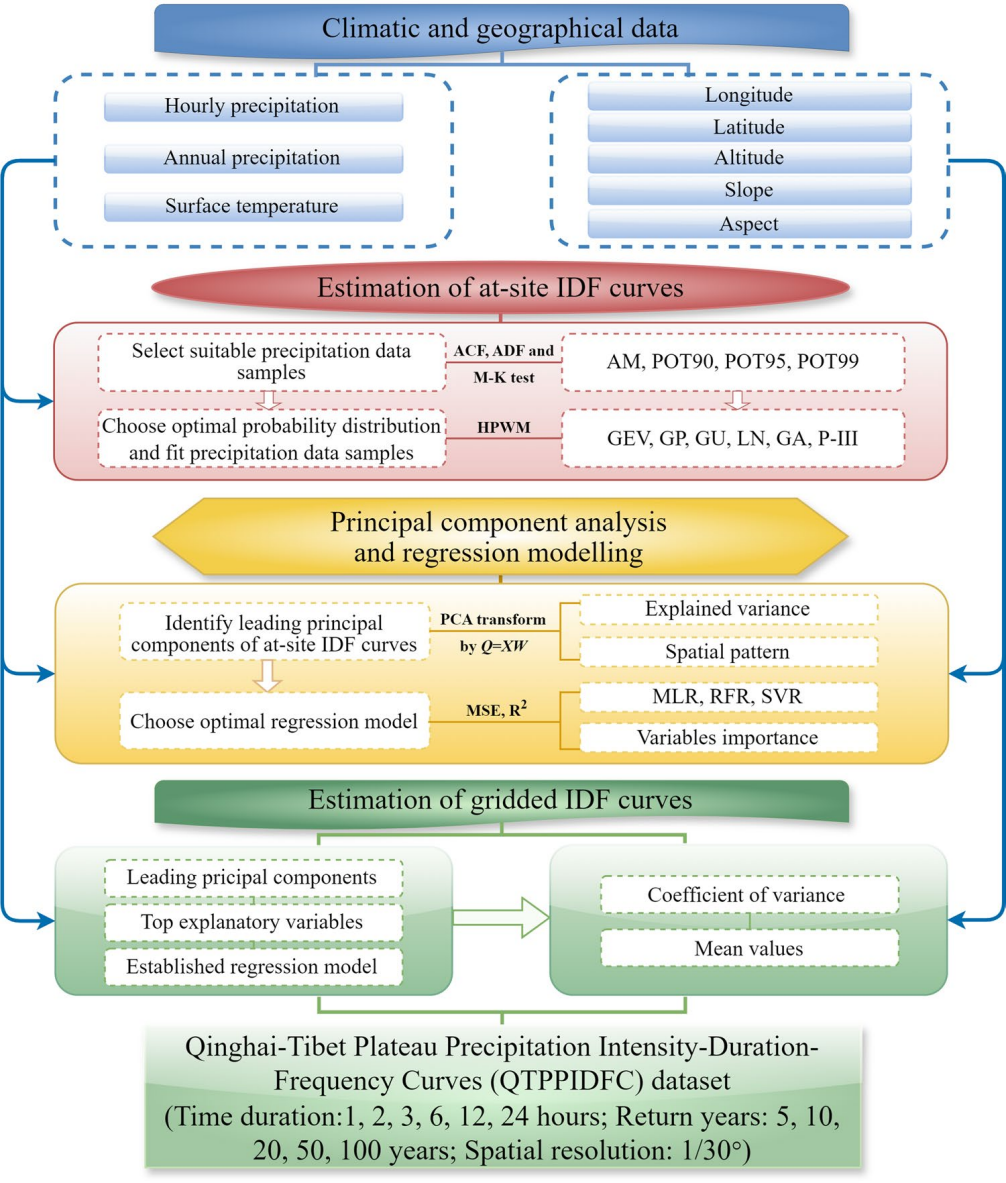
Workflow of generating the QTPPIDFC dataset in this study.

The workflow of generating the QTPPIDFC dataset includes four main steps (Fig. 1):

1. Collect and pre-process precipitation observations and geographical and climatic data;
2. Choose a suitable probability distribution to fit and estimate at-site precipitation IDF curves at each station;
3. Apply the PCA method to identify the spatial pattern and the dominant PCs of all at-site precipitation IDF curves, and establish a regression model to describe the relationship between PCs and geographical and climatic factors;
4. Estimate the gridded precipitation IDF curves covering the whole QTP using the established regression model, and generate the gridded QTPPIDFC dataset.

Each of the four steps in this workflow is explained as below.

### Data collection and pre-processing

#### Hourly precipitation data

The boundary data for QTP^28^ is obtained from the National Tibetan Plateau Data Center (http://data.tpdc.ac.cn). Hourly precipitation data during rainy seasons (May to September) from 1951 to 2018 at 245 weather stations in and around QTP are collected from the National Meteorological Information Center of China Meteorological Administration (http://data.cma.cn/). All the precipitation data have been archived under a standardised procedure to ensure their reliability for scientific research. As the derivation of precipitation IDF curves requires reliable precipitation observations, the data quality at all these stations are further checked. The stations with less than 10 years of precipitation records and with more than 2 years of missing data are excluded. After checking, a total of 203 weather stations are selected for this study, with an average of 38 years of precipitation records.

**Fig. 2:**
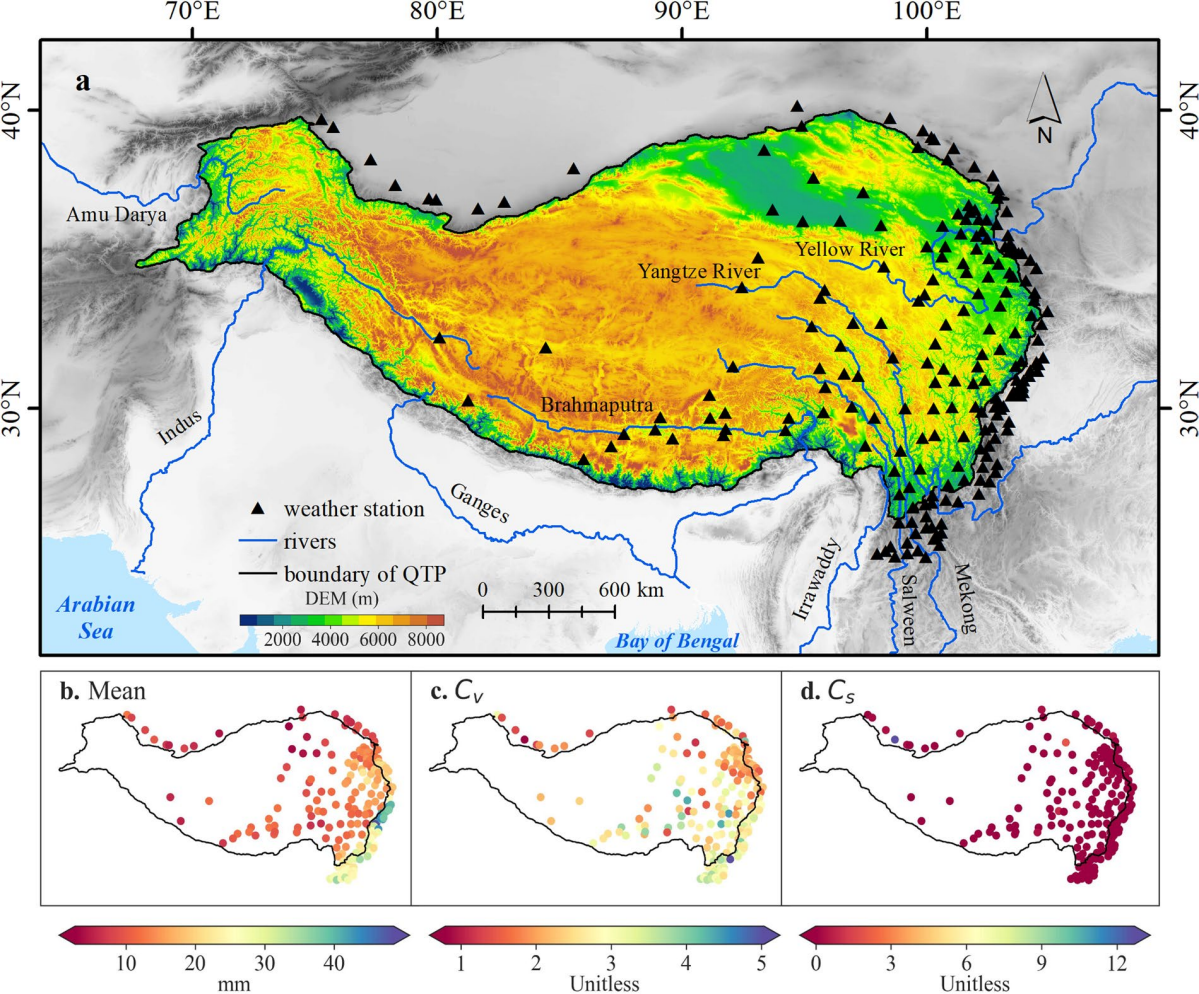
Qinghai-Tibet Plateau (QTP) and locations of the 203 weather stations (**a**) used for this study, and the statistical characteristics of mean value (**b**), coefficient of variance (*C*_*v*_, **c**) and coefficient of skewness (*C*_*s*_, **d**) of annual maximum hourly precipitation at these stations.

The locations of the 203 stations are shown in Fig. 2, with 109 stations located within the interior QTP and 94 stations situated along the rim of QTP. The GTOPO30 Digital Elevation Model (DEM) dataset is used to exhibit the altitudes of QTP and its surrounding regions, which is freely downloaded through the website of USGS (https://www.usgs.gov/). The altitudes of these stations range from 455 to 5094 m.a.s.l., and the statistical characteristics of annual maximum hourly precipitation at these stations are also shown in Fig. 2. Most of the stations are located in the eastern, south-eastern, and southern parts of the region, from where several famous Asia rivers originate, such as the Yellow River, Yangtze River, Mekong River, Salween River and Brahmaputra River. Towns and population are primarily dispersed along rivers in the region, and they are prone to rainstorm hazards, which can cause severe flooding, landslides, and other natural disasters that pose significant threats to the safety and livelihoods of inhabitants.

#### Geographical and climatic variables

We consider geographical and climatic variables to explore the dominant factors influencing the spatial pattern of precipitation IDF curves. Five geographical variables, including longitude, latitude, altitude, slope and aspect at each station, are extracted from GTOPO30 DEM. Two climatic variables are collected, including average annual precipitation (AP_station_) and average daily surface temperature (TEM). The AP_station_ is calculated based on the collected hourly precipitation data. The TEM is calculated based on daily surface temperature observations from 1951 to 2018, which is obtained from daily meteorological dataset of basic meteorological elements of China National Surface Weather Station (V3.0)^29^.

We further collect gridded precipitation data from TPHiPr dataset^30^ with a spatial resolution of 1/30°, considering that the TPHiPr dataset has high accuracy in QTP and is superior to other precipitation data sources in this region^31,32^. The gridded daily precipitation data is used to calculate the AP_TPHiPr_ from 1979 to 2018 at each gird, as an important variable for the spatial interpolation of gridded precipitation IDF curves in the study area.

### Estimation of at-site IDF curves

#### Selection of precipitation data samples

We set the time duration in precipitation IDF curves as *t* = 1, 2, 3, 6, 12, 24 hours, as extreme precipitation events and resulting flooding disasters in QTP mainly occurred at sub-daily scales^33,34^. Here we apply both the annual maxima (AM) sampling method and the peaks over threshold (POT) sampling method to generate extreme precipitation data samples. The AM method takes the annual maximum *t*-hour precipitation as samples; the POT method takes the *t*-hour precipitation over thresholds of 90%, 95% and 99% percentiles as samples. Thus, all the AM, POT90, POT95, POT99 precipitation data samples are obtained.

These four precipitation data samples are further tested, to check if they follow the hypothesis of independent observations^35^, as the prerequisite for fitting probability distributions. We detect (1) possible temporal dependence by the autocorrelation function (ACF) test, with the null hypothesis of independence characteristics, (2) possible temporal monotonic trend by the Mann-Kendall (M-K) test, with the null hypothesis of no monotonic trend, and (3) stationarity by the Augmented Dickey-Fuller (ADF) test, with the null hypothesis of non-stationarity^36^. All the statistics tests are done at the 5% significance level.

**Fig. 3:**
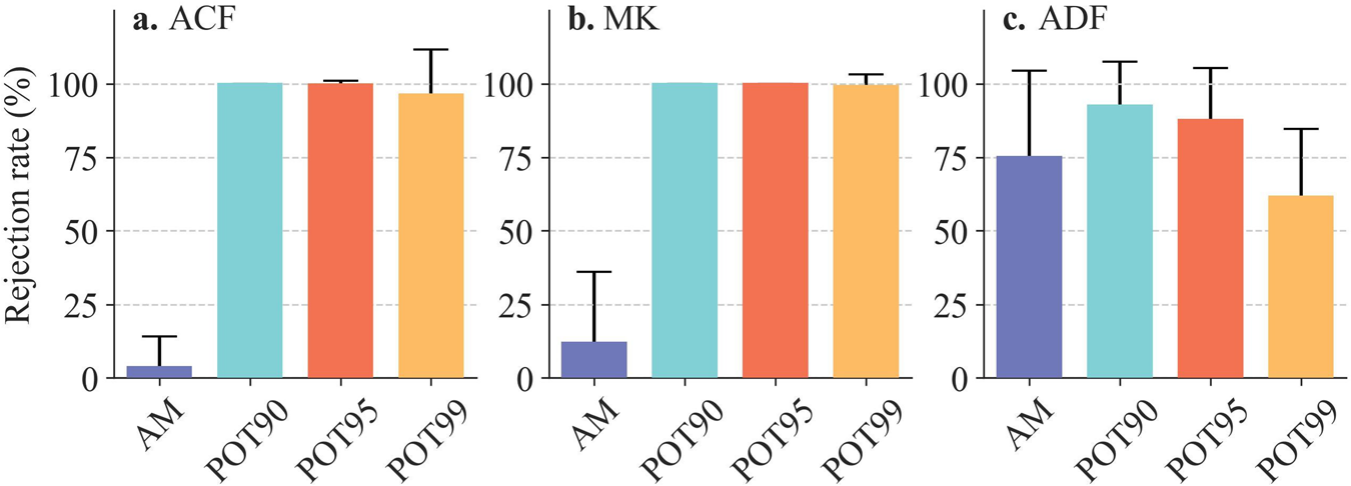
Rejection rate of the null hypothesis (“independence”, “no monotonic trend”, “non-stationarity”) for lag-1 ACF test (**a**), M-K test (**b**), and ADF test (**c**). The length of error bar is equal to the standard deviation of the rejection rate.

As presented in Fig. 3a, the AM precipitation data samples exhibit insignificant lag-1 autocorrelation, with the rejection rate of lag-1 ACF test close to 0%. In contrast, the POT90, POT95 and POT99 precipitation data samples display strong autocorrelation, with a rejection rate close to 100%, indicating an obvious temporal dependence of these POT precipitation data samples. Similar results are also found in lag-2 to lag-4 ACF test results (Supplementary Fig. S1). The results of the M-K test indicate a weak monotonic trend in the AM precipitation data samples, with a rejection rate below 12% (Fig. 3b). Differently, the three POT precipitation data samples exhibit significant monotonic trends, as evidenced by their high rejection rates exceeding 99%. According to the results of the ADF test (Fig. 3c), all precipitation data samples have a relatively high percentage of stationarity. The rejection rate of the AM precipitation data samples is about 75%; the rejection rate for the POT90, POT95 and POT99 precipitation data samples is 93%, 88% and 62%, respectively. Following the above test results, the extreme precipitation data samples with obvious temporal dependence and monotonic trends cannot be used for this study. After comparison, the AM precipitation data samples are selected to estimate at-site precipitation IDF curves.

#### Fitting of precipitation data samples

We consider six probability distribution types used widely in the field of hydrology, and compare them for choosing the most suitable one to fit the AM precipitation data samples. They include generalized extreme value (GEV) distribution^37,38^, generalized Pareto (GP) distribution^38^, Gumbel (GU) distribution^38,39^, lognormal (LN) distribution^40^, gamma (GA) distribution^13,40,41^, and Pearson type III (P-III) distribution^42^.

**Fig. 4:**
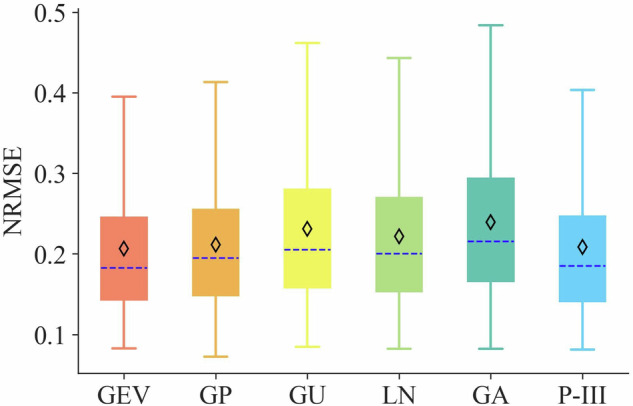
NRMSE of fitting results of AM precipitation data samples by using six probability distributions. Noted that the numbers of samples are not same due to failure in fitting, that is, a total of 1218 samples for GEV, GP, GU, LN, GA distributions, and 1195 samples for P-III distribution. The dashed line and diamond sign represent the median and mean value, respectively.

We use the index of Normalized Root Mean Squared Error (NRMSE) to evaluate the goodness of fit from each probability distribution. The higher-order probability weighted moments (HPWM) method is used for estimating parameters, aiming to improve the estimation of tails of probability distribution, as its superiority compared to other conventional parameter-estimation methods have been verified^43^. Results in Fig. 4 show that the GEV distribution exhibits the best performance in fitting the AM precipitation data samples, with a mean NRMSE of 0.21, indicating its adequacy in capturing the statistic characteristics of AM precipitation data samples. It is noteworthy that the P-III distribution fails in capturing 2% (i.e., 23 out of 1218) of AM precipitation data samples, even with a mean NRMSE of 0.21 for the remaining samples (Fig. 4). A visual check also indicates that the AM precipitation data samples are right-skewed distributed, which is consistent with the GEV distribution (Supplementary Fig. S2). Therefore, the GEV distribution is selected to fit these AM precipitation data samples.

Given the relationship between return year (*T*) and probability (*P*_*r*_), there is:

1 $$T=\frac{1}{{P}_{r}(x\ge {x}_{t,T})}=\frac{1}{1-{P}_{r}(x\le {x}_{t,T})}=\frac{1}{1-F({x}_{t,T})}$$

where ${x}_{t,T}$ is the quantile of precipitation (unit: mm) for *t* duration (unit: hour) and *T* return years. $F({x}_{t,T})$ is the distribution function of GEV, with ${x}_{t,T}$*~*$F(x,k,\xi ,\alpha )$:

2 $$F\left(x,k,\xi ,\alpha \right)=\left\{\begin{array}{ll}{e}^{{-e}^{-(x-\xi )/\alpha }}, & k=0\\ {e}^{{-[1-k\left(x-\xi \right)/\alpha ]}^{1/k}}, & k\ne 0\,{and}\,{k}(x-\xi )/\alpha  < 1\end{array}\right.$$

where $\xi$ is the location parameter, $\alpha$ is the scale parameter, and $k$ is the shape parameter.

### Estimation of at-site precipitation IDF Curves

We consider the return years of 5, 10, 20, 50, 100 years. For each return year, we estimate the corresponding quantiles of *t*-hour precipitation (*t* = 1, 2, 3, 6, 12, 24 hours) using the fitted GEV distribution, and obtain the at-site precipitation IDF curves. Besides, as the parameters are estimated separately on each *t*-hour duration, precipitation at a long duration may be smaller than precipitation at a short duration, causing crossing phenomena between different IDF curves^15^. Therefore, a visual adjustment is done to eliminate these crossing phenomena, and further to ensure the logical consistency and appropriateness of all precipitation IDF curves across diverse durations.

**Fig. 5:**
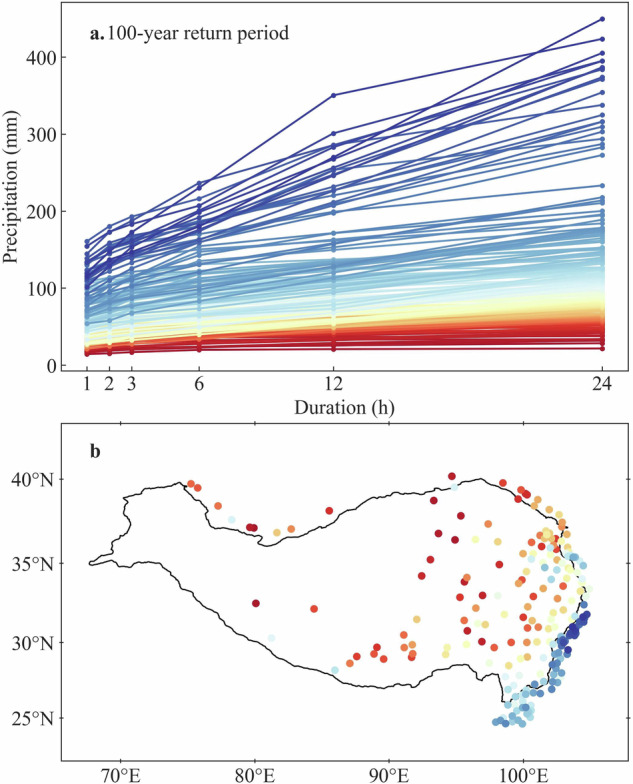
The estimated precipitation IDF curves corresponding to 100-year return period at 203 stations (**a**), and the colours represent stations with different locations (**b**).

These at-site precipitation IDF curves exhibit pronounced spatial patterns, reflecting regional variability in precipitation characteristics. We take the 100-year precipitation IDF curves (Fig. 5a) as an example, they depict an obvious southeast-northwest spatial pattern (Fig. 5b), which should be controlled by the geographical and climatic conditions.

### Principal component analysis

Since the at-site precipitation IDF curves contain substantial precipitation information from hourly to daily durations and corresponding to diverse return years, we apply the PCA method to extract their spatial pattern^26^. When applying the PCA method, the principal components (denoted as *Q*) of all these at-site IDF curves (denoted as matrix *X*) can be described as^44^:

3 $$Q={XW}$$

where *X* is matrix of precipitation amount *x*_*ij*_ with *m* rows (*i* = 1, …, *m*) and *n* columns (*j = *1, …, *n*); for this study *m* = 203 for representing all stations, and *n* = 30 for representing six durations (i.e., *t* = 1, 2, 3, 6, 12, 24 hours) multiplying by five return periods concerned; *W* is a matrix of weights containing eigenvectors of the covariance matrix *X*^*T*^*X*; *W* has *n* rows and its columns is set as 8 (i.e., *k* = 1, …, 8) for this study. The matrix *Q* refers to PCs relevant to matrix *X*, and the *k*th PC = *X×W*_*1:n*_,_*k*_; that is, Q has *n* rows and 8 columns, corresponding to eight PCs. The optimal *W* is determined when the variance in *Q* gets maximum.

**Fig. 6:**
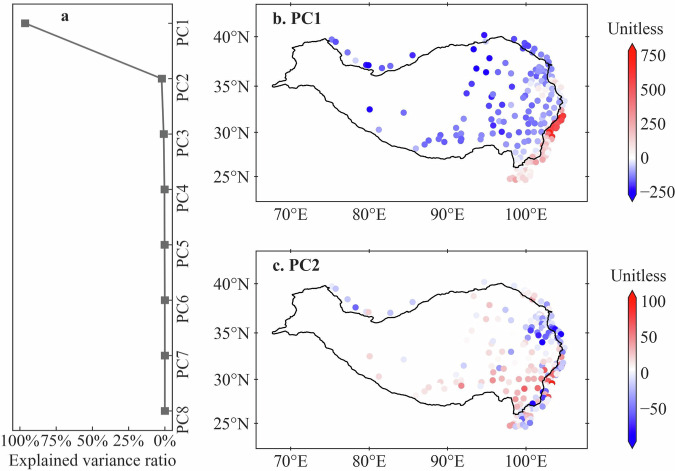
Explained variance ratios (**a**) of eight principal components extracted from all at-site precipitation IDF curves by using the PCA method, and spatial patterns of the PC1 (**b**) and PC2 (**c**).

Figure 6a shows the explained variance ratios of the eight PCs of at-site precipitation IDF curves. The first PC (PC1) contributes to IDF curves with a high 96% explained variance, followed by the second PC (PC2) with a 2% explained variance. Other six higher-order PCs can be taken as noise, as they have small explained variances less than 2% in total. Thus, the first two leading PCs are chosen to reflect the spatial patterns of all at-site precipitation IDF curves. Moreover, as shown in Fig. 6b, PC1 clearly indicates a southeast-northwest spatial pattern, being consistent to the results in Fig. 5b, which indicates the notable explaining strength of PC1 for spatial pattern of at-site IDF curves. On the contrary, PC2 exhibits a descending spatial pattern from southeast to northeast (see Fig. 6c) in the interior QTP.

### Regression modelling

The multiple linear regression (MLR) model, random forest regression (RFR) model, and support vector regression (SVR) model are applied to establish the relationship between the first two PCs of at-site IDF curves and geographical and climatic variables, as the basis of deriving IDF curves from stations to the entire QTP. The three models are employed due to their capability of exploring possible linear or non-linear relationships, as well as their satisfactory performance of dealing with small data samples^45–48^. For MLR model, we apply the ordinary least squares to estimate the parameters. For RFR and SVR models, we use validation-curve to determine the parameters range and then adopt the Optuna algorithm^49^ to obtain the best parameters.

The five geographical variables (longitude, latitude, altitude, slope, aspect) and two climatic variables (AP_station_, TEM), are used as explanatory variables in the three models. The longitude, latitude and altitude mainly determine the geographical conditions of the spatial features of two leading PCs. The two variables of slope and aspect often work as topographical conditions for shaping precipitation by redistributing the water vapor in mountainous areas. The precipitation and temperature are used to test the potential impacts of climatic conditions on the spatial features of two leading PCs.

**Fig. 7:**
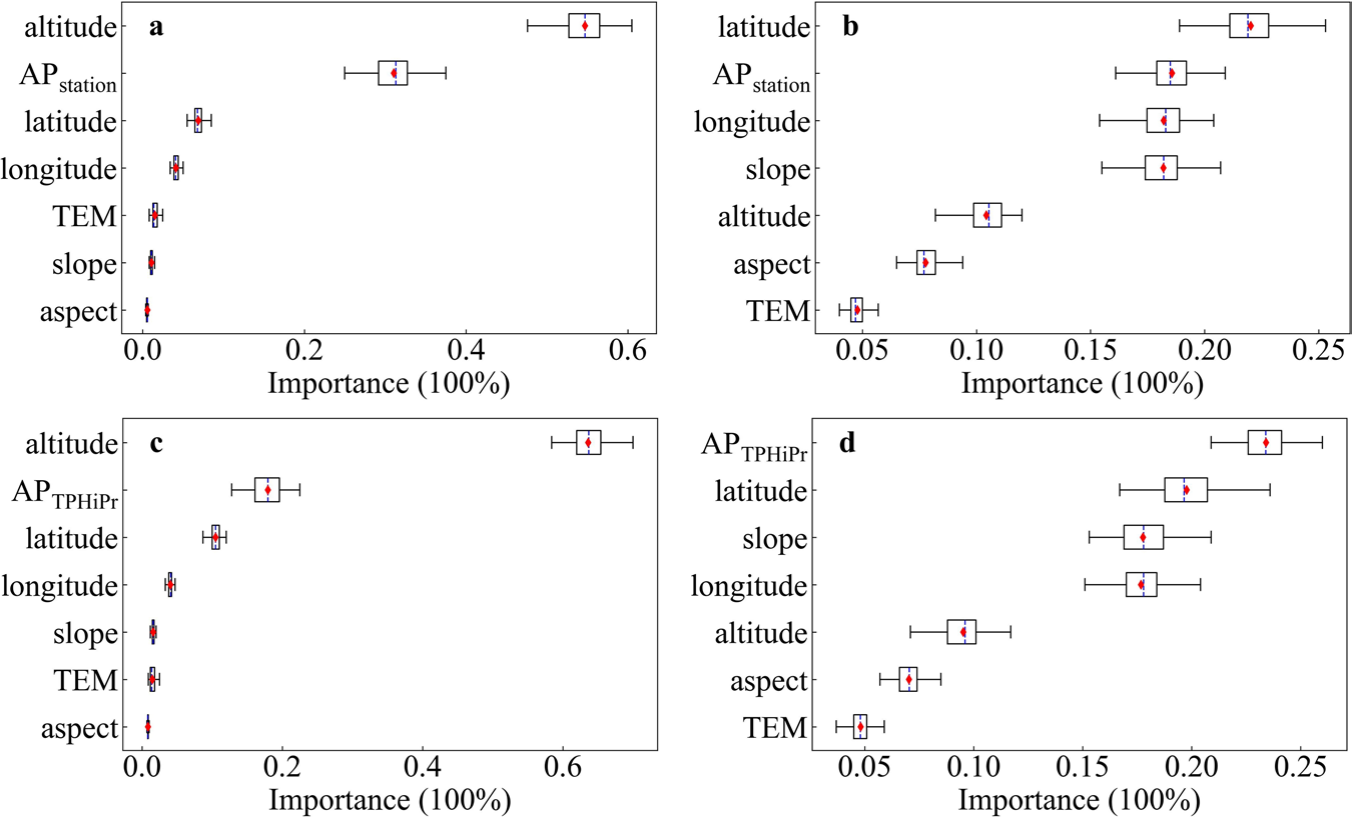
Variable importance evaluated by the RFR model for explaining PC1 (a and c by using AP_station_ and AP_TPHiPr_, respectively) and PC2 (b and d by using AP_station_ and AP_TPHiPr_, respectively). The dashed line and diamond sign represent the median and mean value, respectively.

The variable importance is evaluated by the RFR model. For PC1, the top two important variables are altitude and AP_station_, with a total proportion of 86% importance (Fig. 7a), implying the comprehensive influence of geographical and climatic factors on PC1. The possible reason is that the effect of altitude gradient on precipitation variability is highly significant in mountain areas, thus directly impacting extreme precipitation characteristics. Similar results appear when substituting AP_TPHiPr_ for AP_station_ (Fig. 7c). For PC2, the four variables, namely latitude, AP_station_, longitude, and slope, account for a total of 76% importance (Fig. 7b). When substituting AP_TPHiPr_ for AP_station_ (Fig. 7d), the orders of top four variables changed to AP_TPHiPr_, latitude, slope, and longitude, suggesting the major impact of climatic factors (i.e., AP_TPHiPr_) on PC2.

**Fig. 8:**
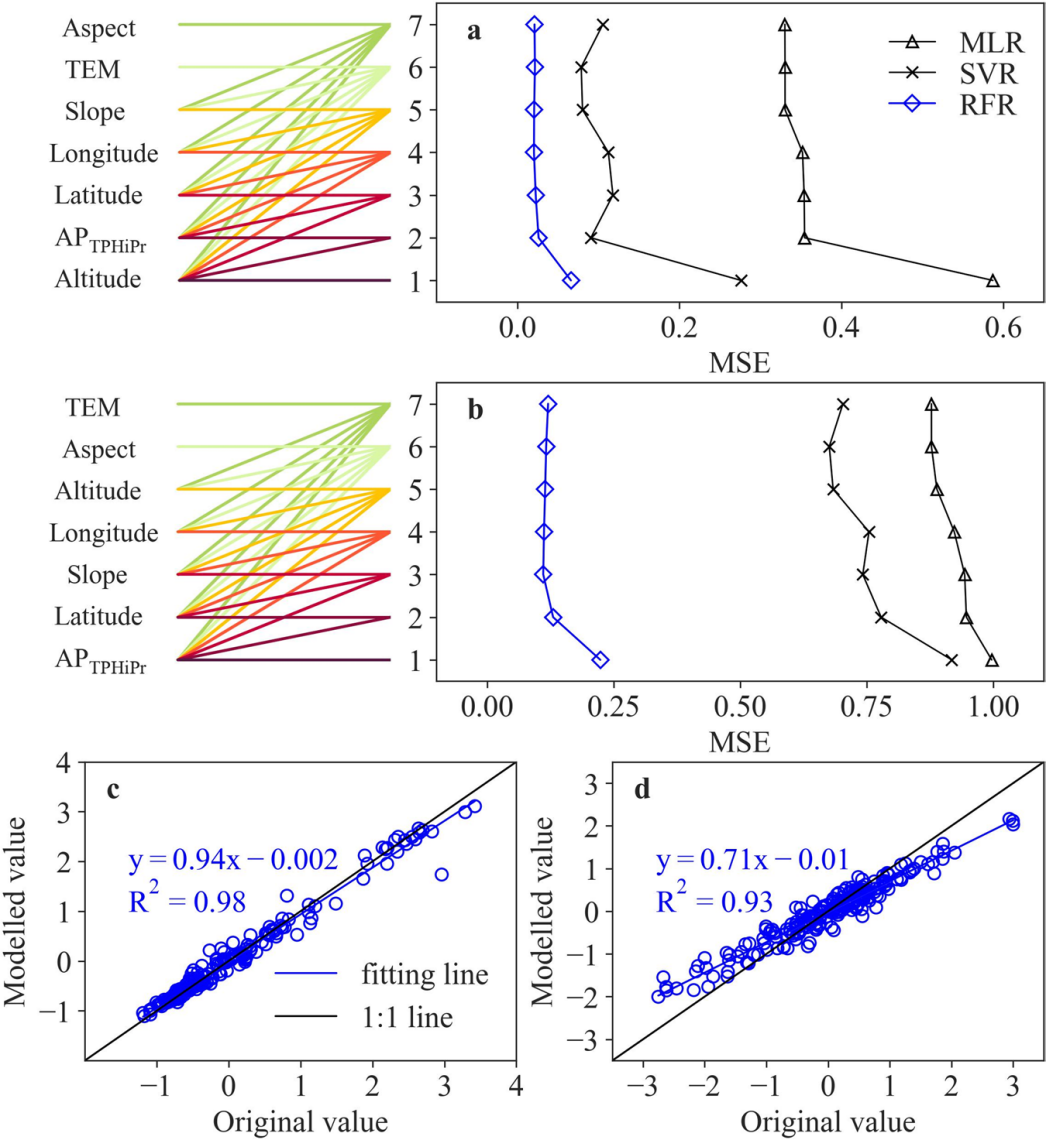
Modelling results of normalized PC1 (**a**) and normalized PC2 (**b**) by using different numbers of top variables in the MLR, SVR and RFR models; and modelling results of normalized PC1 (**c**) and normalized PC2 (**d**) by using top two variables in RFR model. In (**a,b**), the number of 1~7 on ordinates represents the different numbers of top variables used for modelling; for each number of top variables, the corresponding lines have the same colour.

The optimal variables used in regression models are further determined based on the order of the variables’ importance. The modelling accuracy is quantified by using the indexes of Mean Squared Error (MSE) and coefficient of determination (R^2^). Results show that the RFR model outperforms the SVR and MLR model for both PC1 (Fig. 8a) and PC2 (Fig. 8b). It may be due to the notable benefits of the RFR model in terms of reducing overfitting and improving description accuracy of non-linear relationship, while the other two models cannot achieve it. In RFR models, the use of the top two variables significantly improves the modelling accuracy of PC1 and PC2, demonstrating their primary importance in modelling process. However, the modelling accuracy only has slightly improved when adding more variables. Given the RFR model with top two variables, the normalized PC1 can be well modelled by using the altitude and AP_TPHiPr_, with MSE as 0.02 and coefficient of R^2^ as 0.98 (Fig. 8c). However, the normalized PC2 is underestimated when modelled by using the top two variables (AP_TPHiPr_ and latitude), as the MSE is as big as 0.12, although R^2^ is 0.93 (Fig. 8d), which may result from the inadequate explanation from these variables. Thus, considering the weak contribution of PC2 and its modelling difficulty, we use PC1 for the reconstruction of precipitation IDF curves and its derivation at regional scales.

**Fig. 9:**
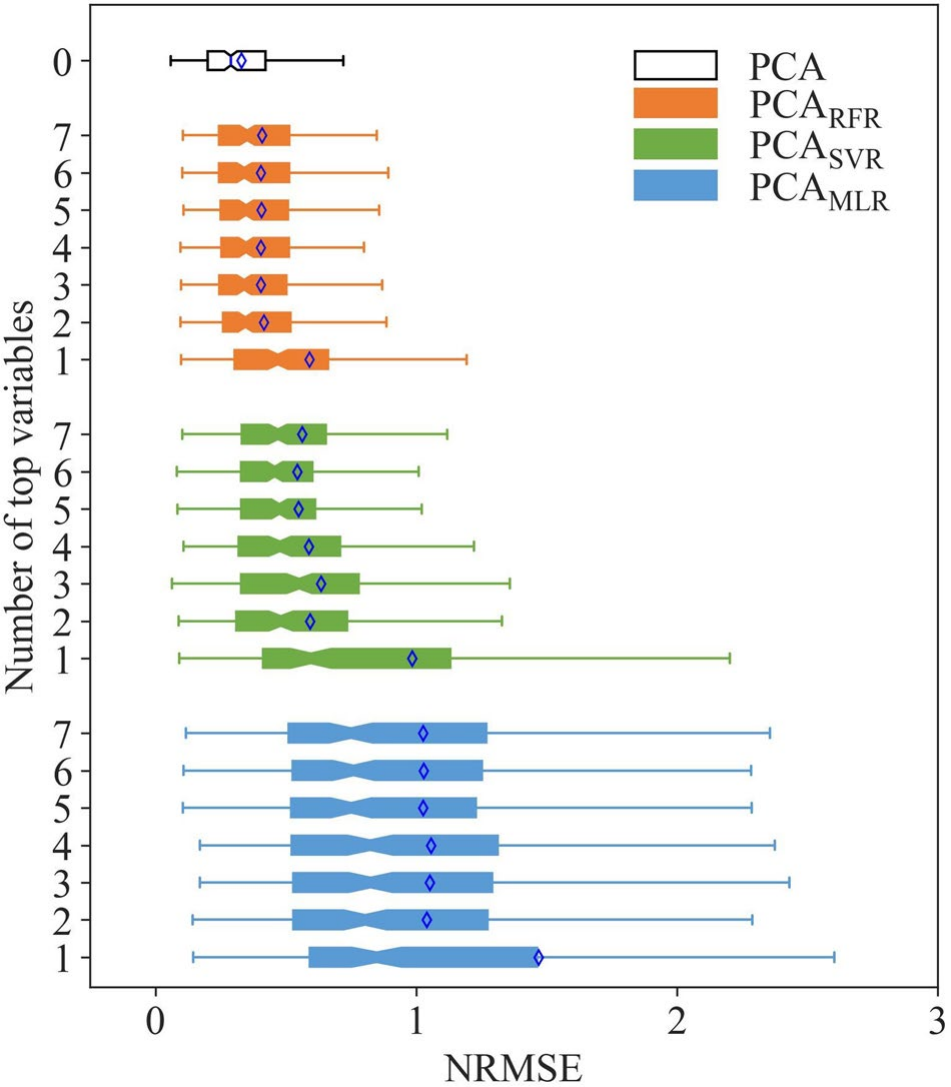
Accuracy of IDF reconstruction by using regressed PC1 with top variables. The number of zero on ordinates represents IDF reconstruction from original PC1, and 1 to 7 represents number of top variables used in the multiple linear regression (PCA_MLR_) model, random forest (PCA_RFR_) model, and support vector regression (PCA_SVR_) model. The number of 1~7 on ordinates has the same meaning as that in Fig. 8a. The diamond sign represents mean value.

Based on the above modelling results of PCs, we reconstruct at-site IDF curves following the established regression-based relationship between PC1 and geographical and climatic variables. As shown in Fig. 9, RFR model outperforms MLR and SVR models in terms of IDF reconstruction, and the accuracy of IDF reconstruction gets improved when using top two variables, which are consistent with the results of modelling PCs in Fig. 8. In RFR model, the IDF reconstruction by using regressed PC1 with top two variables (altitude and AP_TPHiPr_) has a mean NRMSE of 0.42, which is close to direct reconstruction by using original PC1 (0.33), implying the reliability of the modelling results.

As a result, considering the performances of both the models and the variables, the RFR model for PC1 is established, employing altitude and AP_TPHiPr_ as predictors for the spatial derivation of gridded precipitation IDF curves in QTP.

### Estimation of gridded IDF Curves

In order to make a spatial derivation of IDF curves to the entire QTP, we collect gridded AP_TPHiPr_ with a spatial resolution of 1/30°, which are calculated from the TPHiPr dataset, and the gridded altitude, which are extracted from GTOPO30 DEM by resampling tools (to 1/30° spatial resolution) on ArcGIS platform, for modelling PC1 by using the established RFR model. The modelled PC1 are further used to estimate gridded precipitation IDF curves in QTP. To quantify the estimation uncertainty arising from inherent randomness in RFR model’s predictions, we repeat the modelling process for 100 times. This iterative approach enables us to assess the mean value and coefficient of variance (*C*_*v*_) of the gridded precipitation IDF curves, thereby providing a more robust assessment of their uncertainty.

**Fig. 10:**
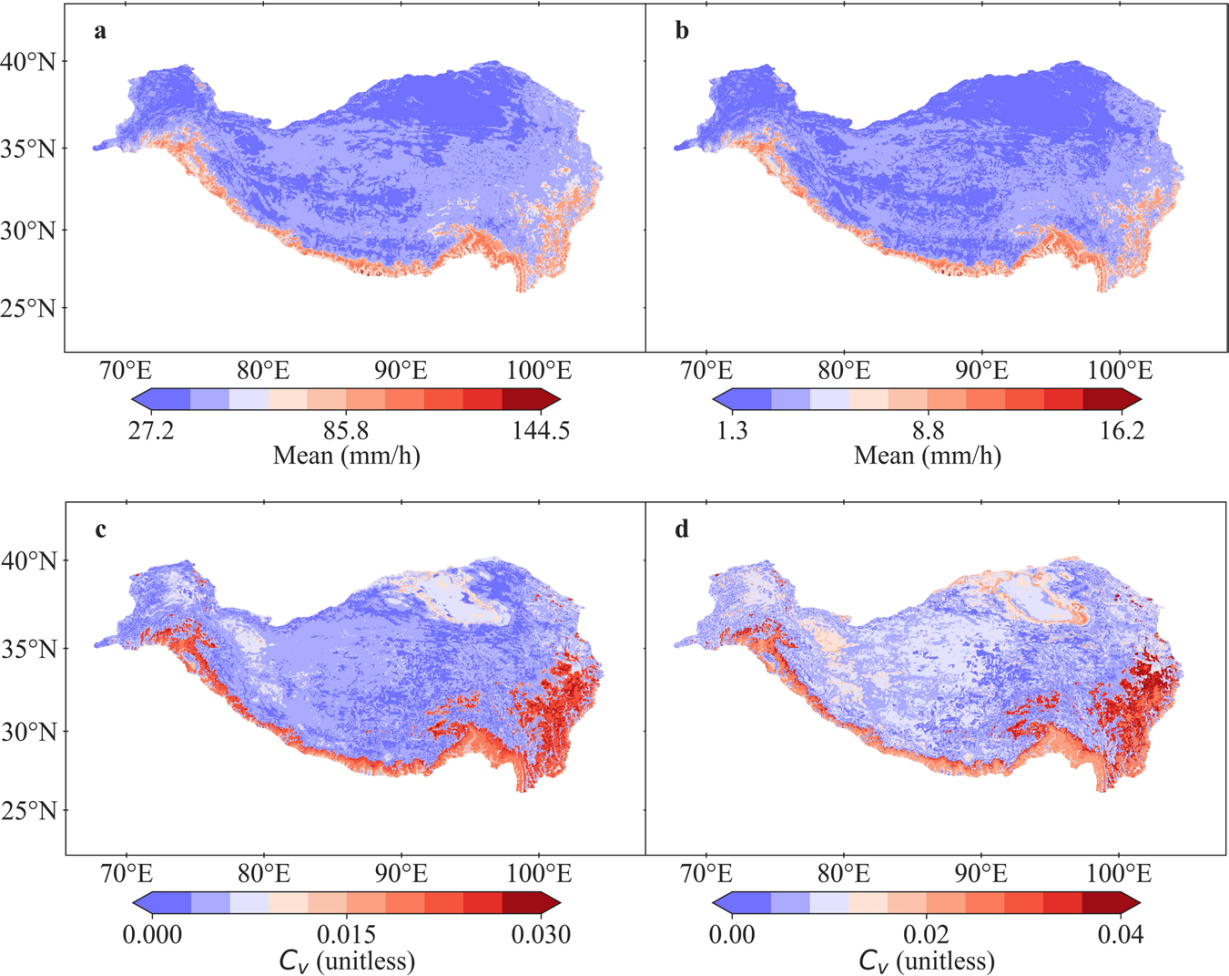
Spatial distribution of the mean value of estimated gridded hourly precipitation intensity (**a**) and daily precipitation intensity (**b**), and their coefficient of variance (*C*_*v*_) values (c and d, respectively) in 100 return years in QTP, with a 1/30° spatial resolution.

Based on the spatial derivation of IDF curves, the QTPPIDFC dataset is generated, which could supply gridded (1/30° spatial resolution) *t*-hour precipitation (*t* = 1, 2, 3, 6, 12, 24) in 5, 10, 20, 50, 100 return years covering the whole QTP. As illustrated by the gridded hourly and daily precipitation intensity in 100 return years (Fig. 10), hourly precipitation intensity has a value range of 27.1~144.5 mm/h, and daily precipitation intensity has a value range of 1.3~16.2 mm/h. Both hourly and daily precipitation intensity exhibit distinct southeast-northwest gradients, with high values appearing in southern boundaries (i.e., Himalaya Mountains) and the southeast part of QTP. The *C*_*v*_ of both hourly and daily precipitation intensity have a similar spatial distribution as that of the mean value. Moreover, it should be noticed that the values of *C*_*v*_ remain small, indicating weak uncertainty and implying the reliability of the QTPPIDFC dataset generated.

### Performance metrics

In this research, a set of performance metrics is employed to assess the disparity between original values (${y}_{i}$) and modelling values (${\hat{y}}_{i}$). Specifically, the Normalized Root Mean Squared Error (NRMSE) is utilized as a quantitative measure to evaluate the goodness of fit of a probability distribution, and the accuracy of the PCA method. The Mean Squared Error (MSE) and coefficient of determination (R²) are utilized to evaluate the performance of validation in three regression models. These metrics are described as:

4 $${\rm{NRMSE}}=\frac{\sqrt{\frac{1}{n}\mathop{\sum }\limits_{i=1}^{n}{({y}_{i}-{\hat{y}}_{i})}^{2}}}{{std}({y}_{i})}$$

5 $${\rm{MSE}}=\frac{1}{n}{\sum }_{i=1}^{n}{({y}_{i}-{\hat{y}}_{i})}^{2}$$

6 $${{\rm{R}}}^{2}=1-\frac{\mathop{\sum }\limits_{i=1}^{n}{({y}_{i}-{\hat{y}}_{i})}^{2}}{\mathop{\sum }\limits_{i=1}^{n}{({y}_{i}-\bar{{y}_{i}})}^{2}}$$

where *std(*$${y}_{i})$$ refers to the standard deviation of $${y}_{i}$$, and $$\bar{{{\rm{y}}}_{{\rm{i}}}}$$ refers to the mean of $${y}_{i}$$.

## Data Records

### Generated dataset

The QTPPIDFC dataset^50^ is publicly available in National Tibetan Plateau Data Center at 10.11888/Atmos.tpdc.301308. The dataset contains two “.txt” files with gridded mean value and *C*_*v*_ of *t*-hour precipitation (*t* = 1, 2, 3, 6, 12, 24) in 5, 10, 20, 50, 100 return years, as well as the longitude and latitude of each grid.

## Technical Validation

### Leave-one-out cross-validation

We do the leave-one-out cross-validation (LOOCV) to evaluate the reliability of the modelling results of PCs. During the validation period, the dependent (i.e., PCs) and predictors (i.e., geographical and climatic variables) of a station are leaved from the modelling calibration. The regression model is subsequently applied to predictors of the left stations to estimate the value of PCs. The LOOCV is accomplished until independent estimates of PCs are obtained for all stations. The accuracy of the model is evaluated by computing the average MSE derived from LOOCV processes, which can ensure the reliability of modelling results. We repeat the LOOCV for 100 times, to obtain the optimal parameters in regression models.

**Fig. 11:**
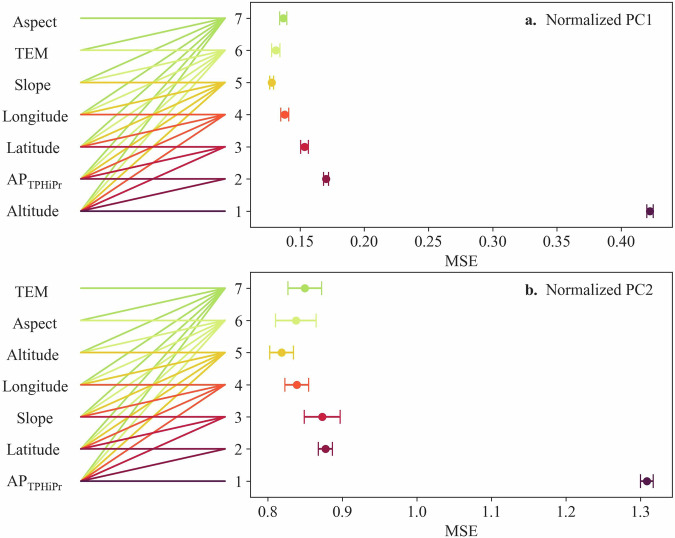
The modelling results of normalized PC1 (**a**) and normalized PC2 (**b**) by using different variables for the leave-one-out validation. The length of the error bar is equal to the standard deviation of MSE. Note that the number of samples to calculate MSE is 202, which is different from that (i.e., 203) in Fig. 8a,b.

Here we present the MSE of 100 LOOCV processes in RFR models (Fig. 11). It shows that the accuracy of models gets improved when using the top two variables, which is consist with the results of modelling PCs (as shown in Fig. 8a,b) and IDF reconstruction (as shown in Fig. 9). The results of LOOCV, as well as the unbiased estimate of PC1 (shown in Fig. 8c), justify the reliable modelling performance and the reliable quality of the generated QTPPIDFC dataset.

## Usage Notes

QTP is well known for its high vulnerability to rainstorm hazards and induced natural disasters. Exploration of extreme precipitation characteristics is crucial for improving hydrometeorological risk management and hydraulic/hydrologic engineering design in the region. In this research, to fill the precipitation IDF data gap, we generate the QTPPIDFC dataset by using the PCA method and the RFR model. The dataset provides gridded precipitation information within 1, 2, 3, 6, 12, and 24 hours corresponding to 5, 10, 20, 50, and 100 return years, with a 1/30°spatial resolution. Overall, this dataset can solidly serve hydrometeorological-related risk management and hydraulic/hydrologic engineering design in QTP.

Finally, it should be noted that the QTPPIDFC dataset is subject to a few limitations:

1. In this research, we mainly focus on natural disasters triggered by intense rainfall, and thus derive the IDF curves from stations to the entire QTP, focusing on rainfall-dominated areas where local population and economic activities are concentrated. In fact, there are other areas covered by ice and snow in QTP, particularly in high-altitude areas with sparse weather stations, and their related natural disasters such as glacial lake outburst floods are far from the key topic of this research and should be studied separately.
2. We use the AM precipitation data samples to estimate at-site precipitation IDF curves. However, the non-stationarity of 25% AM precipitation data samples (as shown in Fig. 3) may affect the results of estimated precipitation IDF curves. To clarify it, we select 22 stations with records exceeding 60 years and consider three periods: the entire period (period-I), the first half of 30 years (period-II) and the last half of at least 30 years (period-III). For the three periods, results in Supplementary Fig. S3 show a relative stable range of mean, standard deviation, and *C*_*v*_, suggesting little influence of non-stationarity in precipitation data samples on the final results. Thus, it is acceptable and feasible to use AM precipitation data samples to derive the gridded precipitation IDF curves covering the entire QTP.

## Supplementary information

Supplementary Information

## Data Availability

The Python codes used for generating the QTPPIDFC dataset^51^ are available at 10.5281/zenodo.13143415.
