# Supplementary material for: A dataset of gridded precipitation intensity-duration-frequency curves in Qinghai-Tibet Plateau
Source: Sci Data. 2025 Jan 2;12:3. doi: 10.1038/s41597-024-04362-1 (PMC11695680; doi:10.1038/s41597-024-04362-1)
Supplement: Supplementary file 1 — Supplementary Information [file 41597_2024_4362_MOESM1_ESM.docx]

### Supplementary Information of “A dataset of gridded precipitation intensity-duration-frequency curves in Qinghai-Tibet Plateau”

**Zhihui Ren^1,2^, Yan-Fang Sang^1,2,3,4,^, Peng Cui^2,5^, Chen Fei^6^, Deliang Chen^7,8^**

^1^Key Laboratory of Water Cycle & Related Land Surface Processes, Institute of Geographic Sciences and Natural Resources Research, Chinese Academy of Sciences, Beijing, 100101, China.

^2^University of Chinese Academy of Sciences, Beijing, 100049, China.

^3^Yarlung Zangbo Grand Canyon Water Cycle Monitoring and Research Station, Tibet Autonomous Region, Linzhi, 860000, China.

^4^Key Laboratory of Compound and Chained Natural Hazards, Ministry of Emergency Management of China, Beijing, 100085, China.

^5^Key Laboratory of Land Surface Pattern and Simulation, Institute of Geographic Sciences and Natural Resources Research, Chinese Academy of Sciences, Beijing, 100101, China.

^6^POWERCHINA Chengdu Engineering Corporation Limited, Chengdu, 610072, China.

^7^Regional Climate Group, Department of Earth Sciences, University of Gothenburg, 40530, Gothenburg, Sweden.

^8^Department of Earth System Sciences, Tsinghua University, Beijing 100080, China

**List of Figures**

[Fig. S1 Rejection rate of the null hypothesis ("independence") for lag-2 (a), lag-3 (b), lag-4 (c) ACF test. The length of error bar is equal to standard deviation of rejection rate. 2](#_Toc182560684)

[Fig. S2 Histogram of AM precipitation data samples and probability density function (PDF) of generalized extreme value (GEV) distribution in an example station. 2](#_Toc182560685)

[Fig. S3 The mean (a, b, c), standard deviation (d, e, f) and *C_v_* (g, h, i) of AM precipitation data samples on three periods of 22 stations with record length longer than 60 years. The dashed line and diamond sign represent the median and mean value, respectively. 3](#_Toc182560686)


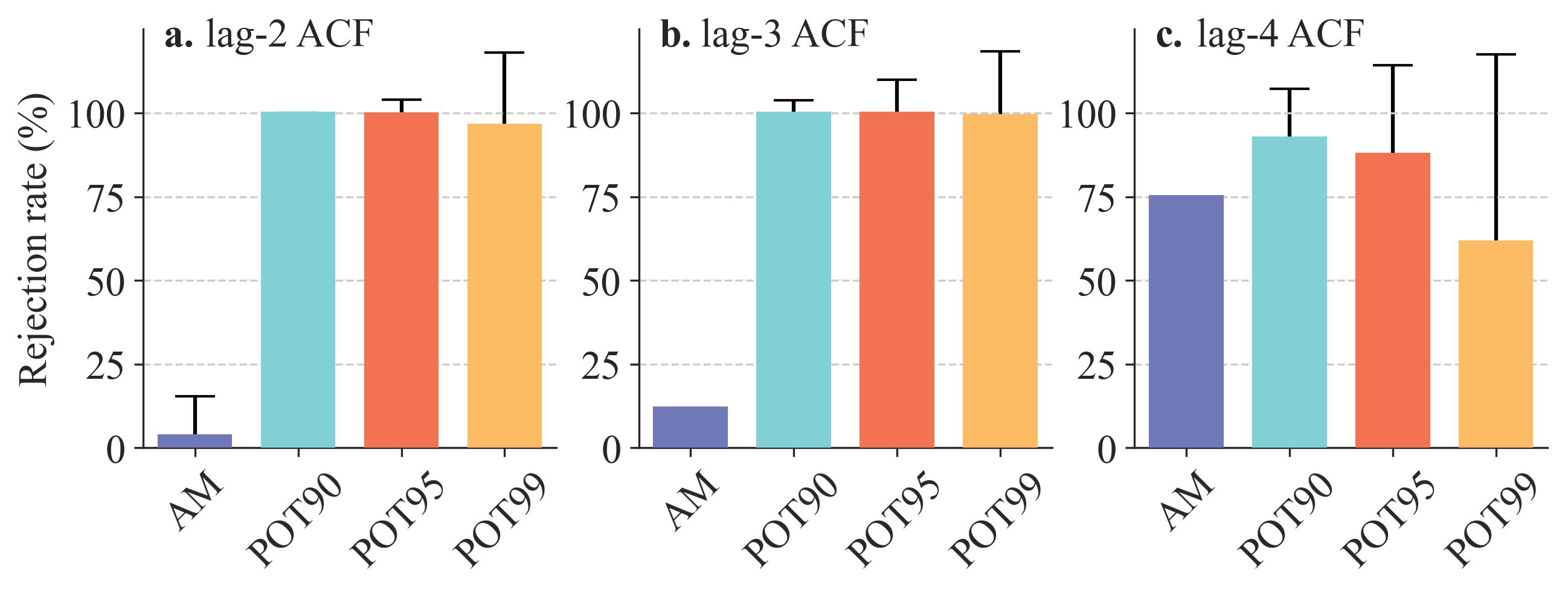


Fig. S1 Rejection rate of the null hypothesis ("independence") for lag-2 (a), lag-3 (b), lag-4 (c) ACF test. The length of error bar is equal to standard deviation of rejection rate.


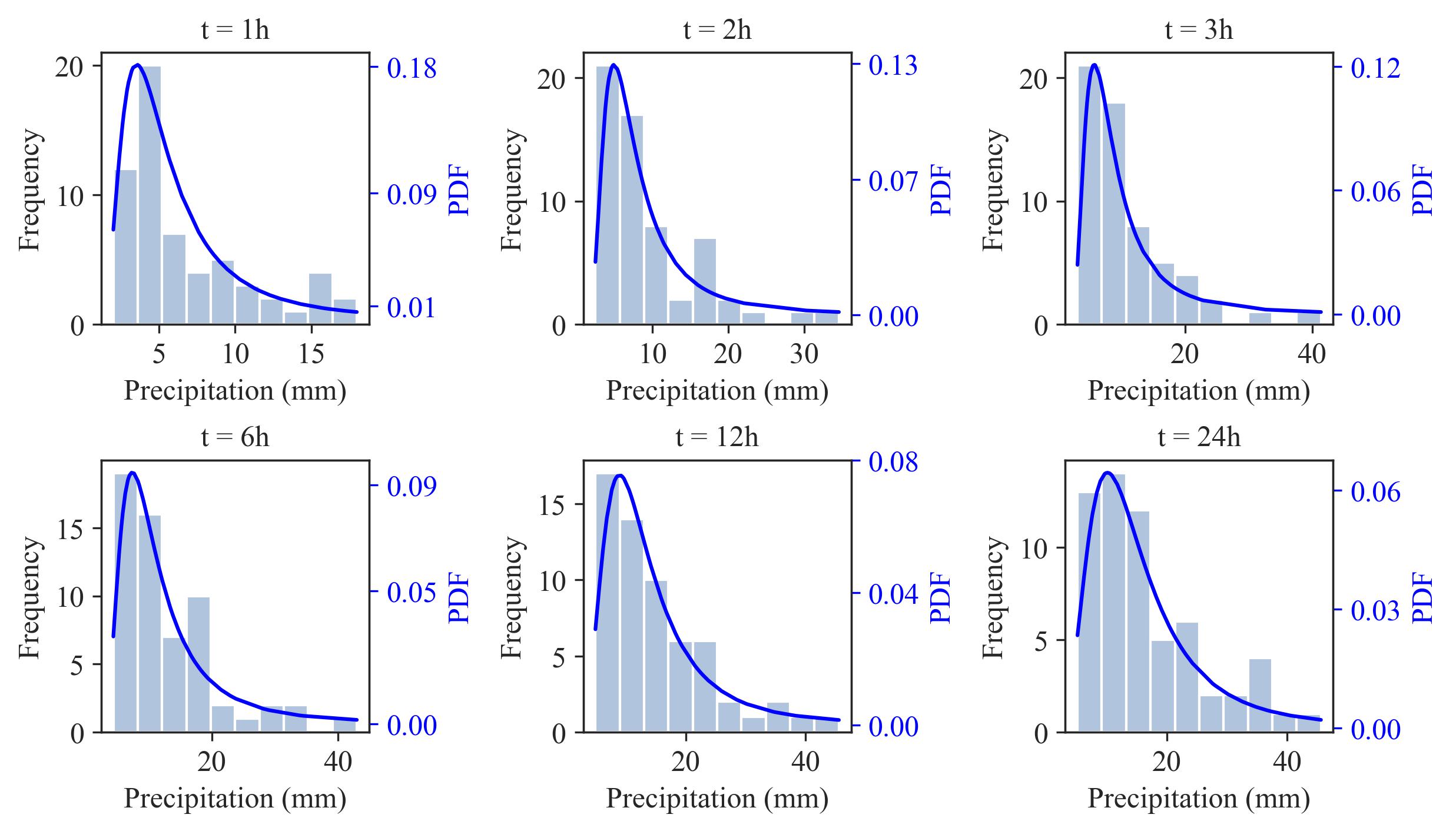


Fig. S2 Histogram of AM precipitation data samples and probability density function (PDF) of generalized extreme value (GEV) distribution in an example station.

**
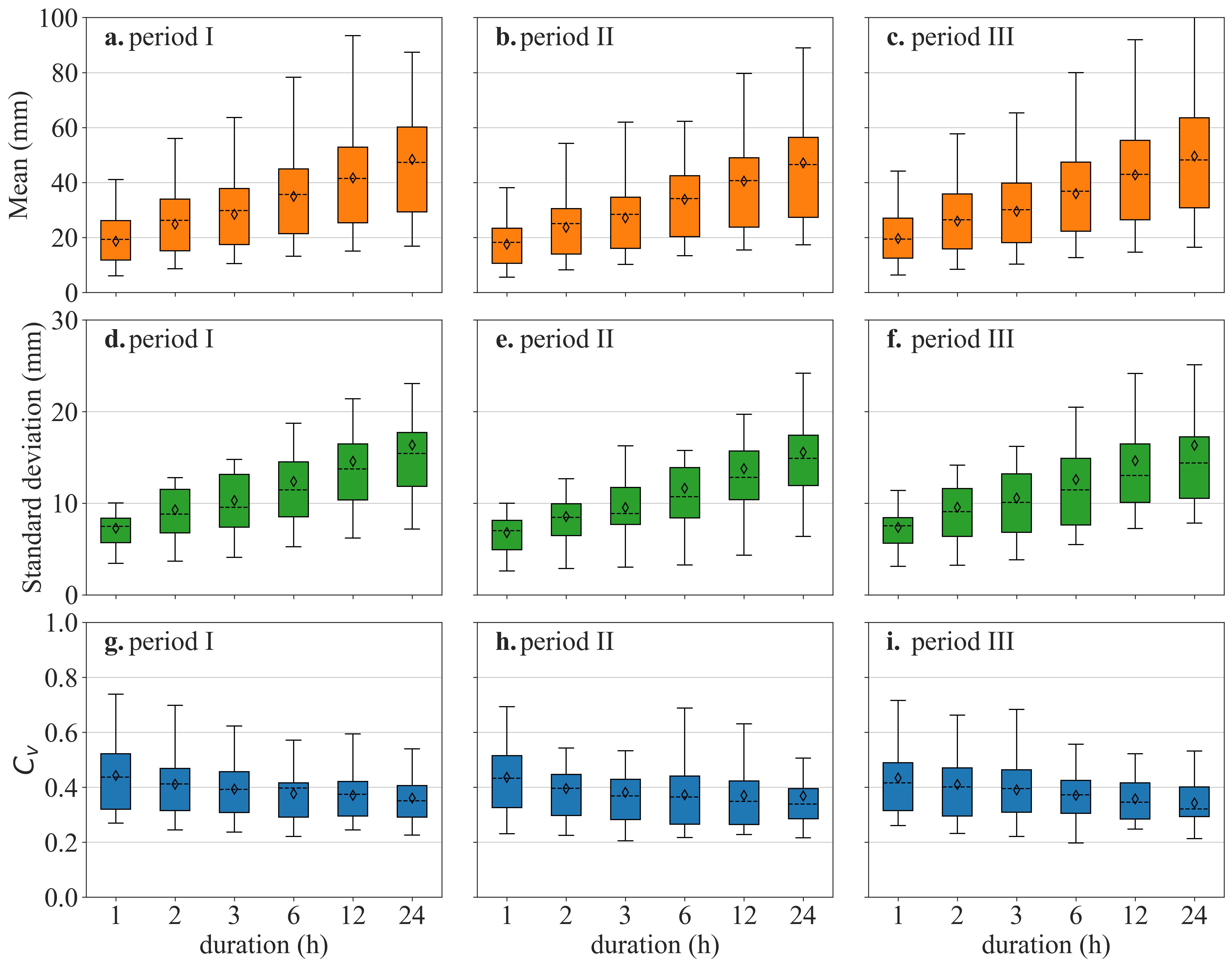
**

Fig. S3 The mean (a, b, c), standard deviation (d, e, f) and *C_v_* (g, h, i) of AM precipitation data samples on three periods of 22 stations with record length longer than 60 years. The dashed line and diamond sign represent the median and mean value, respectively.
